# Early estimation of the epidemiological parameters of novel coronavirus disease (COVID-2019) outbreak in Iran: 19 Feb-15 March, 2020 

**Published:** 2020

**Authors:** Meysam Olfatifar, Walid Q. Alali, Hamidreza Houri, Mohamad Amin Pourhoseingholi, Ebrahim Babaee, Romian Seifollahi, Afsaneh Sharifian, Mohammad Reza Zali

**Affiliations:** 1 *Gastroenterology and Liver Diseases Research Center, Research Institute for Gastroenterology and Liver Diseases, Shahid Beheshti University of Medical Sciences, Tehran, Iran *; 2 *Department of Epidemiology and Biostatistics, Faculty of Public Health, Kuwait University, Safat, Kuwait *; 3 *Foodborne and Waterborne Diseases Research Center, Research Institute for Gastroenterology and Liver Diseases, Shahid Beheshti University of Medical Sciences, Tehran, Iran*; 4 *Preventive Medicine and Public Health Research Center, Department of Community Medicine, Iran University of Medical Sciences, Tehran, Iran*

**Keywords:** COVID-19, Iran, Outbreak, Epidemiological parameters

## Abstract

**Aim:**

To estimate the epidemiological parameters related to the Covid-19 outbreak in Iran.

**Background:**

Estimating the epidemiological parameters of new public health threat (COVID-19) is essential to support and inform public health decision-making in different communities including Iran.

**Methods:**

We established a mathematical model to estimate the epidemiological parameters from 19 Feb to 15 March based on daily COVID-19 confirmed cases in Iran. Then, we estimated the effect of early traffic restriction on our estimation.

**Results:**

We estimated the R0 at 2.11 (95% CI, 1.87-2.50) and the infected number at 92,260 (95% CI: 59,263 -152,212) by 15 March. Our estimate for the ascertainment rate was about 1.2% (95% CI: 1.1-1.4). The latent period estimation was 4.24 (95% CI: 2.84-6.65). We observed a decline in our estimate after considering the traffic restriction.

**Conclusion:**

Our results suggest that health authorities in Iran must take impactful strategies to control the COVID-19 outbreak to reach R0<1. Therefore, the establishment of complementary, multilateral, and cost-effective measures for the treatment of symptomatic and early diagnosis and isolation of asymptomatic cases/contacts are strongly recommended because of low ascertainment rate and large number of infected cases. We additionally recommend that traffic restriction be combined with other controlling measures.

## Introduction

 COVID-19 was firstly reported in Dec 2019 by Chinese health authorities (1) and has now been labeled as a pandemic by WHO and considered a global health emergency (2). However, the scientific gaps about the disease are being filled, and now the important role of person-to-person transmission (3) and subsequently asymptomatic transmission as a potential source of the outbreak (4) have been addressed. 

Notably, health authorities in some affected countries are taking important decisions with incomplete and evolving scientific data, which highlights the importance of identifying and estimating epidemiological parameters such as the basic reproductive number (R_0_)(5), ascertainment rate, latent period, and the number of infected people to understand the true extent of the outbreak.

Hence, estimating these parameters are crucial to take a suitable and timely response to this outbreak. In countries such as Iran, current epidemiological data are insufficient to understand the true epidemiological picture of this disease and the complexity of its epidemic (6). Additionally, COVID-19 has affected all the provinces in Iran, and hence we urgently need to estimate these epidemiological parameters.

The objective of this study was to estimate the epidemiological parameters related to the Covid-19 outbreak in Iran by establishing a mathematical model owing to its ability to guide control strategies. 

## Methods

**Domestic connectivity of Qom province**

**Figure 1:**
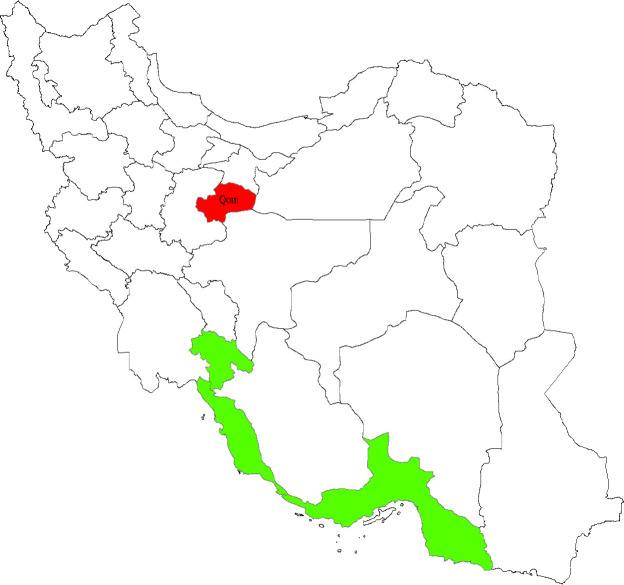
The connectivity of Qom (red polygon) with other provinces directly (white polygons) or indirectly (green polygons) by other provinces, either by airline or landline

On 19 Feb, the first confirmed cases of COVID-19 were reported in Qom province, Iran (Figure 1, red polygon). Qom is a religious city with direct contact with all provinces of the country (Figure 1, white polygons) except for Hormozgan, Bushehr, and Kohgiluyeh and Boyer-Ahmad provinces (Figure1, green polygons).

In this modeling study, we considered Qom province as the source of infection dissemination to other provinces. This project has been ethically approved by the Ethics Committee in Gastroenterology and Liver Diseases Research Center, Research Institute for Gastroenterology and Liver Diseases, Shahid Beheshti University of Medical Sciences, Tehran, Iran. (NO.1106)

**Mathematical modeling and statistical analysis**

In this study, we used embedded functions from the Wuhan package (7, 8) in R software to estimate the epidemiological parameters of the Covid-19 outbreak in Iran. Briefly, a deterministic SEIR metapopulation transmission model implemented in the Wuhan package was used to estimate the transmission rate, latent period, infected number, ascertainment rate, and basic reproductive number. 

**Model Assumptions**

First, we assumed that the daily number of infected cases has Poisson distribution based on the following equation:

$y_{\mathrm{is}}\mathrm{\sim Possion}(\emptyset_{i}x_{\mathrm{is}})$

Where $y_{\mathrm{is}}$ is the number of newly detected cases in each Iranian province “i” for each day “s” (9). Similar $x_{\mathrm{is}}$ is the number of expected for each province for a specific day and $\emptyset_{i}$ is the province-specific case ascertainment between 0 and 1. The case trajectory in this SEIR (susceptible, exposed, infected, and recovered) model was constructed based on ordinary differential equations. 

In this way, the force of infection for each province of Iran is 

$\lambda_{t}=\beta (I/N+(K\cdot (I_{t}/N))/N)$

β is the human-to-human transmission rate, *k* is the connectivity matrix between the Iran provinces where each element represents the number of daily passengers that move between the provinces (either by airline or landline) (10, 11), *I* is the number of infected people, and N is the population number of each province. 

Then, by constructing the log-likelihood function and its optimization (maximization), we estimated the aforementioned epidemiological parameters (7, 8). In the final step, we used the bootstrap to consider the uncertainty around the estimated parameters by calculating the 2.5% and 97.5% quantiles.

We only included COVID-19 daily cases from 12 Feb to 15 March and generalized our results based on that time period. Our assumption preceded the implementation of domestic travel limitations and lockdown in most Iran provinces that received a significant number of infected cases. Furthermore, although the number of domestic travels considerably increased around March 15 due to the Iranian New Year’s Eve holiday, we assumed that the daily number of passengers remained constant in that time period. Finally, we quantified the impact of travel restriction on COVID-19 by a 20% reduction in the number of daily passengers. 

## Results

**Figure 2:**
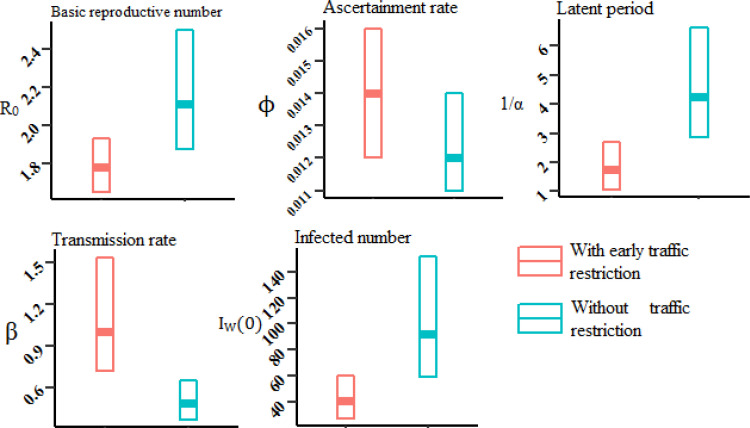
Estimated epidemiological parameters of COVID-19 in Iran, with and without considering traffic restriction

Regarding the importance of COVID-19 and to obtain better epidemiological facet, we estimated the disease epidemiological parameters in Iran and showed the impact of early traffic restrictions. We estimated the basic reproductive number (Ro), from 19 Feb to 15 March at 2.11 (95% CI, 1.87-2.50). Moreover, by considering a 20% reduction in the number of daily passengers from reporting the first confirmed cases in Iran, the Ro decreased to 1.77 (95% CI, 1.65-1.93) (Figure 2).

The latent period estimation was 4.24 (95% CI, 2.84-6.65) and with the 20% traffic reduction, it dropped to 1.76 (95% CI, 1.07-2.68) (Figure 2). The average ascertainment rate was estimated approximately at 1.2% (95% CI, 1.1-1.4), and with 20% traffic restriction, it was diminished to 1.4% (95% CI, 1.2-1.6) (Figure 2).

Our estimation for the number of infected people was at 92,260 (95% CI, 59,263 -152,212) and 40,630 (95% CI, 27,454-60,066) without and with traffic restriction, respectively (Figure 2). Finally, our estimate for the transmission rates was 0.49 d^-1 ^(95% CI, 0.37-0.66) and with 20% traffic restriction it was 1.00 d^1 ^(95% CI, 0.72- 1.54). These results could be an indication of the positive effect of traffic restrictions on the control of the Covid-19 outbreak.

## Discussion

We estimated the epidemiological parameters of Covid-19 for Iran. The estimated reproductive number of 2.11 (95% CI, 1.87-2.50) is reasonably consistent with other reports in other regions of the world mostly in China (12-15) and approximately consistent with the higher bound of WHO estimate of 1.4-2.5. Meanwhile, our estimate was less than other reports that estimate R_0 _around 3 or higher (16, 17). However, the time fluctuation of this estimate during an outbreak is quite expected. Therefore, one evidence showed that R_0_ estimates first increase and then approach its initial values (18). Therefore, we believe that our Ro estimate is reasonable at this time, 15 March. When considering early traffic restrictions from the beginning of the outbreak in Iran, we observed about 0.3 declines in the R_0_ estimate. However, it should be interpreted cautiously because one evidence indicates that the impact of traffic restriction on the spread of the COVID-19 outbreak is modest unless combined with other public health measures (19).

Likewise, we estimated the number of infected cases at about 92 thousand cases. The projected higher number than the currently reported confirmed cases could be explained by the presence of a large number of asymptomatic peoples and those who are under 14 years of age. This may indicate that most patients in Iran are asymptomatic and could potentially be contagious to others. One evidence in nature publisher supports our results well, stating that these asymptomatic cases can account for about 60% of all infections (20). Furthermore, the role of asymptomatic contacts has previously been approved in Germany(21).

Meanwhile, it is also quite logical that early travel limitations can lead to a smaller number of infected people traveling to each province. However, this measure should be implemented along with other control measures. 

Our estimate for the ascertainment rate was less than other active surveillance systems in other countries where ascertainment rate was at 9.2%(22) or 44% for non-severe cases(23). Nevertheless, various reasons can be involved such as asymptomatic cases and mild illness(22). Accordingly, we strongly recommend implementation of an active screening using more reliable and valid screening methods such as PCR-based diagnostic tests to identify symptomatic and asymptomatic cases since past screening methods such as temperature checks to rule out cases are not reliable(20). Likewise, our estimate of ascertainment rate can support the positive role of travel limitation on the COVID-19 outbreak.

There are some limitations to our work. First, Poisson increment in the number of cases as this increment might have another distribution. Second, we did not consider the effect of intervention programing in our model other than the traffic restriction. Third, the daily fluctuations in the number of passengers were not included in our model. In the meantime, these results can guide to implement interventions against COVID-19 in Iran.

In general, our model has estimated important epidemiological parameters and showed the positive impact of travel restriction among provinces in Iran on the estimated number of cases. Future models could take other social distancing measures and other public health interventions into considerations.
